# Facilitating a More Diverse Workforce: The Value of Mentorship in Cardiothoracic Surgery

**DOI:** 10.1016/j.atssr.2024.06.008

**Published:** 2024-06-25

**Authors:** Tanisha Rajah, David Blitzer, Hannah Copeland, Hiroo Takayama

**Affiliations:** 1 Birmingham Medical School, University of Birmingham, Birmingham, United Kingdom; 2 Division of Cardiac, Thoracic, and Vascular Surgery, Department of Surgery, NewYork-Presbyterian/Columbia University Irving Medical Center, New York, New York; 3 Division of Cardiothoracic and Vascular Surgery, Lutheran Hospital Indiana University School of Medicine–Fort Wayne, Fort Wayne, Indiana

## Abstract

**Background:**

Cardiothoracic surgery (CTS) is one of the least diverse surgical specialties in both gender and race. Aside from the inherent benefits of a diverse working environment, mitigating this diversity gap improves patient care. Mentorship is important for creating a diverse, nurturing environment for trainees. This review appraises the impact of formal mentorship for trainees and specifically assesses its significance for underrepresented groups in CTS.

**Methods:**

A literature search was performed using keywords relating to CTS, mentorship, and underrepresented groups. Principal findings were extracted and synthesized; supporting literature discussing mentorship in other surgical specialties was also included.

**Results:**

Mentorship is desired by CTS residents. Its benefits include aiding the matching process, decreasing stress, and increasing academic productivity. Mentorship programs can be used to increase the recruitment and retention of women and others underrepresented in medicine.

**Conclusions:**

Mentorship is a valuable tool for which implementation must be prioritized in CTS; this requires more education on mentorship for trainees. Institutions should be using mentorship programs to diversify trainees in CTS with the objective being equality.

## Visual Abstract

**Figure:**
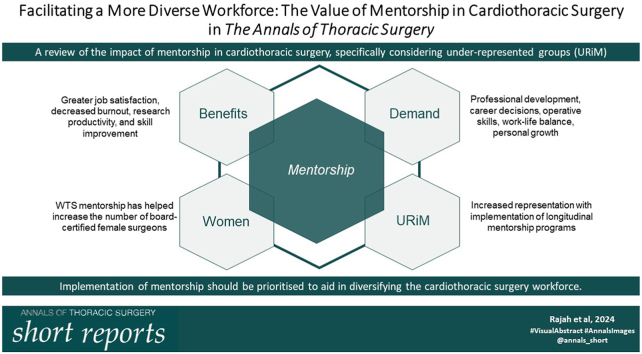

**In Short**

▪ Mentorship benefits cardiothoracic surgery residents by aiding the matching process, decreasing stress, and increasing academic productivity.
▪ Cardiothoracic surgery lacks diversity, particularly concerning both gender and race. Mentorship programs can help facilitate a more diverse working environment for trainees.

Surgical education was built on the basis of apprenticeship. This has translated to the development of mentorship in the modern era.^1^ By definition, mentoring is a 1-to-1 relationship between an expert or experienced individual and a trainee, with the expert voluntarily giving up time to teach, support, and encourage the protégé.^2^ Whilst its definition may appear clear-cut, in reality, mentorship remains somewhat elusive or evolving based on the mentee’s needs throughout their professional journey and personal circumstances. Hence, mentees may have one or multiple mentors concomitantly or at various points during their careers.

Mentorship provides numerous benefits for the career trajectory of mentees, including stress reduction, confidence, positive risk taking, research productivity, skill improvement, and increased resident satisfaction with surgical training.^3^, ^4^, ^5^ Thus, a mentor is deemed a critical element to success by 91% of cardiothoracic surgery (CTS) residents.^4^

Mentorship has recently evolved from an organic connection formed between 2 individuals to a formal structured process aimed at enabling juniors to acclimatize to their chosen speciality and its challenges.^4^ This formal approach can be mutually beneficial, providing professional stimulation and personal enrichment for both mentors and mentees.^6^

This review assesses the prevalence and impact of formal mentorship for CTS trainees. A diverse environment in health care improves patient satisfaction, particularly when patients are treated by providers of the same ethnicity. In addition, gender diversity enhances research knowledge perspectives.^7^ Therefore, the significance of mentorship on underrepresented groups will also be considered.

## Material and Methods

A search of electronic databases including PubMed and MEDLINE was conducted to identify relevant literature for this review between the date of inception and June 2023. Combinations of keywords were used, including *mentorship*, *mentor*, *mentee*, *medical education*, *education*, *surgery*, *cardiothoracic*, *cardiac*, *thoracic*, *CT*, *training*, *residency*, *women*, and *URiM*. Studies included in the search were those that were directly related to mentorship. All studies were limited to texts written in English. Excluded studies included those not carried out in the United States or Canada. Given the limited data on mentorship within CTS, this review provides a narrative overview. However, a systematic review would offer greater value once additional studies are available.

## Results

Of the 431 studies identified through use of the search terms and hand searching, the 7 most relevant studies were included. Of these, 4 studies involving 415 participants focused directly on mentorship in CTS; 2 implemented mentorship models, whereas the other 2 distributed surveys to gauge trainee perspectives. In addition, 3 studies involving 301 participants concentrated on mentorship for women in CTS, with 2 assessing the impact of mentorship programs and 1 exploring social media’s influence on mentorship. These studies are summarized in Table 1 and Table 2, respectively.

**Table 1:** Mentorship in Cardiothoracic Surgery

Study (Year), Journal	Study Objective	Study Design and Participants	Targeted Stage of Training	Program/Survey Evaluation and Results	Study Conclusion
Coyan (2022)^8^ *The Journal of Thoracic and Cardiovascular Surgery*	Evaluating implementation of a protocol to increase residents’ academic productivity in CTS	Mentorship model: dyad; regular research meetings, project prioritization and feedback Mentees: 34 residents Mentors: academically productive faculty	Residents	Program evaluation: pre/postsurvey on academic productivity (peer-reviewed articles, abstract presentations [oral/poster] at national CTS meetings, textbook chapters) Results: 122 peer-reviewed articles produced, abstract oral/poster presentations increased from 11 to 40, textbook chapters increased from 4 to 15	Implementation of a protocol substantially improved the academic productivity of CTS residents
Deivasigamani (2022)^9^ *Journal of Surgical Research*	To understand how structured mentorship affects matching applicants to categorical general surgery programs	Mentorship model: dyad and group; 7 meetings/events during 1 year Mentees: 26 medical students Mentors: senior medical students and senior faculty	Medical students	Program evaluation: institutional match data, student Likert scale responses, and student application data Results: 6 events had median helpfulness scores (out of 5) that were significantly higher than a “neutral” baseline (*P* < .05); 100% matched into a categorical general surgery or integrated subspecialty residency	Structured mentorship programs can be a low-cost implementation at medical schools to aid in the match process
Reich (2021)^10^ *The Annals of Thoracic Surgery*	To evaluate mentorship effectiveness in CTS and to identify gaps in mentorship education	Survey (included trainee experiences, expectations, and perspectives on mentorship) 67 residents	Residents	Survey evaluation: mentorship effectiveness tool (composite score of 0-55; score ≤44 indicating less effective mentorship) Results: 83.6% reported a current mentor, median mentorship effectiveness score was 51, 61.2% of residents had no education on mentorship, 53.8% were not currently a mentor	Addressing gaps in mentorship education and delivery should be prioritized
Stephens (2018)^4^ *The Journal of Thoracic and Cardiovascular Surgery*	To understand the current state of mentorship in CTS training	In-Training Examination survey: traits sought in mentors, impact of mentors, demographics of chosen mentors, areas in which residents lacked mentorship 288 residents	Residents	Survey evaluation: scale used to quantify responses (extremely, moderately, slightly, or not at all important) Results: junior residents valued mentors as role models; senior residents appreciated mentors in technical training, job counseling, and societal involvement; women valued mentors as role models and for networking; men valued mentors for teaching technical skills and clinical ability	The role of mentorship varies with program type, seniority, and gender; therefore, it needs to be tailored to the individual

CTS, cardiothoracic surgery.

**Table 2:** Mentorship for Women in Cardiothoracic Surgery

Study (Year), Journal	Study Objective	Study Design and Participants	Targeted Stage of Training	Program Evaluation and Results	Study Conclusion
Papageorge (2022)^11^ *JTCVS Open*	Assessing the experience of previous WTS mentorship program participants	Mentorship model: dyad 39 participants	Medical students, residents, and practicing cardiothoracic surgeons	Program evaluation: survey Results: the program provided participants with more opportunities, reassurance, belonging, and an available role model rather than changing career trajectory	This intervention can contribute to gender equity in CTS
Williams (2021)^12^ *The Annals of Thoracic Surgery*	Assess the impact of WTS scholarships on career milestones	Mentorship model: dyad 106 WTS scholarship recipients	Medical students and residents	Program evaluation: retrospective data collection and post-scholarship survey Results: 26% of medical students entered integrated CTS residency and 37% entered general surgery residency 59% of general surgery awardees entered CTS fellowships 100% of CTS resident/fellow awardees earned ABTS certification; 44% of ABTS-certified awardees are practicing cardiothoracic surgeons at US academic training institutions 100% reported that their scholarship was valuable in their development	Scholarship recipients pursued CTS career milestones at significantly higher rates than non-recipients
Luc (2018)^13^ *Seminars in Thoracic and Cardiovascular Surgery*	Determining the role of social media in mentorship for women cardiothoracic surgeons	Survey 156 respondents (27 cardiothoracic)	Pre–medical students, medical students, residents, and practicing surgeons	35-item survey: demographics, training and/or professional information, personal and/or family status, career choice decisions, perception of specialty domination by men, access to mentorship, use of social media (personal and professional), effectiveness of social media for mentorship, important factors resulting in successful mentorship experience on social media Results: CTS respondents were more likely to use social media to network and to engage with same-sex mentors compared with other specialties; CTS respondents were more likely to lack exposure to same-sex mentors at their own institution	Social media enhances networking and mentorship, particularly for women in CTS Longitudinal studies on the effectiveness of mentorship by social media are needed

ABTS, American Board of Thoracic Surgery; CTS, cardiothoracic surgery; WTS, Women in Thoracic Surgery.

## Comment

### Mentorship in Cardiothoracic Surgery

Mentorship is valuable in translating initial interest during medical school into pursuing a career in the speciality through increasing exposure to program information and networking,^9^^,^^14^ Trainees are also more likely to choose the same career as their mentor.^13^ Sidney Kimmel Medical College conducted a structured 1-year program for students interested in applying to general surgery, integrated plastics, vascular, or cardiothoracic residencies. This entailed 7 standardized meetings/events across the students’ third and fourth years involving reviewing curriculum vitae, personal statements, mock interviews, and discussion of long-term career goals. All participants who applied to a general surgery residency were matched into a categorical program, surpassing the national match rate of 73%. Two of the 26 successful applicants matched into integrated CTS programs. Implementation of mentorship proved to be a cost-effective strategy in aiding the match process.^9^

Only some CTS residencies have formal mentorship programs in place.^4^ Despite the low prevalence of mentorship programs, studies demonstrate that it is desired by CTS residents.^15^ Reasons include professional development, career decisions and operative skills, personal skills including work and life balance, clinical confidence, and personal growth.^10^

A study by Reich and associates^10^ involved distribution of an anonymous survey to CTS trainees in Accreditation Council for Graduate Medical Education–accredited programs to evaluate mentorship effectiveness and to highlight gaps in mentorship education. Of 531 invited trainees, 67 participated. Most respondents (83.6%) had a current mentor, with subjective median mentorship effectiveness being 51 of 55 (a score of 44 or lower indicated less effective mentorship). A third of residents described having no mentor, less effective mentorship, or both. In addition, 61.2% of residents had no education on how to act as an effective mentor; hence, 53.8% of residents were not currently partaking as a mentor.^10^

The perceived most valuable characteristic of a mentor varied with experience, program type, and gender of the resident^10^; 75.8% stated being “easy to work with and approachable” as the most important aspect of mentor selection, followed by “mentor was in subspecialty I was interested in” and “mentor was studying area of interest in research.” Frequently reported reasons for not having a mentor included “cannot identify someone who truly reflects what you need,” “time constraints,” and “unsure how to get mentor.”^10^ Hence, lacking aspects of mentorship also differ by the specific needs of the mentee.^4^

There was no association between resident age, gender, race or ethnicity, marital status, family status, postgraduate year, and training program type or size with having a mentor. However, because of a low survey response rate of 12.6%, the statistical validity of the findings may be limited.^10^ This review’s findings confirm that although many residents have mentors, there is still a gap, resulting in ineffective mentorship or no mentorship taking place for some residents. Residents with mentors may have been more inclined to partake in the survey, resulting in response bias. The study reflects the need for more structured education on mentorship for trainees.

At the level of an individual institution, the University of Pittsburgh implemented faculty mentorship to increase the academic productivity of CTS residents through regular research meetings. All measured outcomes of academic productivity per resident numerically increased after implementation: number of peer-reviewed articles (0.6 vs 4.1; *P* = .01), abstract presentations (oral/poster) at national meetings (0.6 vs 1.3; *P* = .01), and textbook chapters (0.2 vs 0.5; *P* = .01). Conclusively, the implementation of this structured cardiac surgery research program significantly increased the academic productivity of the resident physicians.^8^

### Mentorship for Underrepresented Groups in Cardiothoracic Surgery

CTS remains one of the least diverse specialities, with insufficient numbers of women and those underrepresented in medicine (URiM).^16^ URiM is defined by the Association of American Medical Colleges as racial and ethnic populations that are underrepresented in the medical profession relative to their proportion in the general population.^17^ The systematic review of Hemal and coworkers^18^ concluded that longitudinal mentoring programs were the most effective strategy to increase the presence of women and URiM in surgery.

Women make up <10% of all practicing surgeons^11^; in 2017, only 5% of American Board of Thoracic Surgery–certified surgeons were female despite more than half of medical students being female.^2^^,^^19^ Role models and networking through mentorship are more valued by women, possibly given the increased importance of relationships to women or because men have been “unofficially” mentored throughout residency for years.^4^ Hence, mentorship can be used to aid in the recruitment of women surgeons.

Established in the United States in 1986, the Women in Thoracic Surgery (WTS) organization aims to provide mentorship to female surgeons during their training.^11^ Their mentorship program matches undergraduate students, medical students, residents, or attendings interested in CTS with female surgeons in the field.^11^ The program has achieved a 70% recommendation rate of participants and is highly valued for career development.^11^^,^^12^

The efforts of the WTS have increased work satisfaction and have contributed to the increased number of board-certified female surgeons from 2012 to 2020.^20^ These targeted endeavors have set a paradigm for regions such as Europe, where a committee for women in CTS was only established in 2021, reflecting a positive change in recruiting women into the speciality.^20^

Social media may facilitate mentorship for women. It can enhance networking and provide more exposure of female trainees to mentors by overcoming the geographic distinction often associated with residency training.^13^^,^^21^

It was thought that women required same-sex mentors to overcome challenges unique to women.^22^ However, Farkas and coworkers^23^ found that gender-discordant vs concordant pairs did not determine mentorship satisfaction, indicating that this is not a barrier to implementation. Thus, mentorship can by implemented by mentors from a variety of backgrounds.

There is insufficient representation of URiM in CTS, and those URiM that do decide on CTS are less likely to have a mentor both as a trainee and as faculty.^24^^,^^25^ The US population consists of 60% white, 19% Hispanic, 13% Black, and 2% Native American or Hawaiian, yet this translates to comprising 66% White, 8% Hispanic, 4% Black, and 0% Native American or Hawaiian CTS residents.^26^ Targeted mentorship can be used to change this underrepresentation.

To address the lack of URiM in urology, residents at the University of California, San Francisco initiated the UnderRepresented Trainees Entering Residency (UReTER) mentorship program in 2020. The program paired URiM medical students (Black, LatinX, and indigenous students) interested in urology with current urology residents and fellows, providing formal mentorship for residency applications^27^; 71 mentor-mentee pairs (dyad model) were made, each meeting at least 2 times during the application cycle. A post–Match Day survey collected feedback; 16 participants responded, 94% (15) matched into a urology program, and 38% (6) attributed their match to the program. Other mentoring programs targeting URiM in urology have also exemplified early success from the University of California Los Angeles and the University of Michigan.^28^

In 2021, Bonifacino and colleagues^17^ conducted a systematic review of mentorship programs for URiM in academic medicine. There were various models of mentorship; the dyad model was the most frequent and reported high satisfaction ratings. The study included 28 programs, with the University of California’s mentorship program resulting in an increase in 4-year retention of URiM junior faculty from 58% to 80%. Racial/ethnic concordance between mentor and mentee did not have an impact on satisfaction with the mentorship received.

Overall, there is a lack of both women and URiM in CTS. This issue is exacerbated by the absence of targeted mentorship programs.^26^ Institutions should be enforcing mentorship programs specifically for women and URiM focused on the recruitment, retention, and promotion of these groups with the objective being equality.

### Conclusion

Mentorship has an overwhelmingly positive impact on the CTS workforce. However, mentorship availability and approaches are highly variable across medical school and residency and for practicing surgeons. Currently, mentorship is not universally mandated in residency training; only 42% of residents have program-assigned mentors,^4^ and those URiM are even less likely to have a mentor during training and as faculty.^25^ Enhancing mentorship prevalence in CTS requires increased evaluation of current mentorship programs, further implementation of formal mentorship, and education on effective mentoring. This will cultivate a diverse, nurturing environment to enable our residents to have every opportunity for personal and professional success.
